# Beyond HY5: COP1 Posttranslational Control of Anthocyanin Biosynthesis Proteins in Horticultural Crops

**DOI:** 10.3390/plants15040616

**Published:** 2026-02-14

**Authors:** Gabriel Lasmar dos Reis, Agustín Zsögön, Antonio Chalfun-Junior, Lázaro Eustáquio Pereira Peres, Vagner Augusto Benedito

**Affiliations:** 1 Department of Biology, Universidade Federal de Lavras (UFLA), Lavras 37200-900, MG, Brazil; gabriellasmarreis@hotmail.com (G.L.d.R.); chalfunjunior@ufla.br (A.C.-J.); 2 National Institute of Science and Technology on Plant Physiology Under Stress Conditions, Department of Plant Biology, Universidade Federal de Viçosa, Viçosa 36570-900, MG, Brazil; agustin.zsogon@ufv.br; 3 Laboratory of Hormonal Control of Plant Development, Luiz de Queiroz College of Agriculture, Department of Biological Sciences, University of São Paulo, Piracicaba 13418-900, SP, Brazil; lazaro.peres@usp.br; 4 School of Agriculture and Food Systems, West Virginia University, 1194 Evansdale Dr., Morgantown, WV 26506-6108, USA; 5 School of Agricultural and Natural Sciences, University of Maryland Eastern Shore, Princess Anne, MD 21853-1299, USA

**Keywords:** bioactive compound, light, post-translational regulation, ubiquitination

## Abstract

Anthocyanins are widespread specialized metabolites that provide pigmentation and antioxidant capacity, contributing to pollinator and seed-disperser attraction and to plant resistance to diverse environmental stresses. In human diets, anthocyanins are valued for their antioxidant and health-promoting properties. The biosynthetic pathway of anthocyanins is relatively conserved across plant species and is controlled by structural genes that encode the enzymes of the pathway along with regulatory genes, particularly transcription factors. This network integrates developmental and environmental signals, with light serving as a dominant cue: anthocyanins typically accumulate in light-exposed tissues and are repressed in darkness. A key node in this light-dependent switch is CONSTITUTIVE PHOTOMORPHOGENIC 1 (COP1), an E3 ubiquitin ligase that, in the dark, promotes polyubiquitination and proteasome-mediated turnover of positive regulators of anthocyanin production. Although ELONGATED HYPOCOTYL 5 (HY5) is a canonical COP1 target and major activator of anthocyanin biosynthesis, COP1 control of this pathway extends well beyond HY5. Evidence from *Arabidopsis* and multiple horticultural crops, including apple, pear, eggplant, and tomato, indicates that COP1 also regulates anthocyanin accumulation through interactions with additional transcription factors and regulatory modules. Here, we synthesize recent advances in COP1-centered regulation of anthocyanin biosynthesis, with an emphasis on post-translational mechanisms and COP1 targets beyond HY5. We also discuss emerging opportunities to leverage this regulatory axis for nutritional improvement in horticultural species.

## 1. Introduction

Anthocyanins are specialized flavonoid pigments derived from the phenylpropanoid pathway, widely found throughout the plant kingdom. Originally recognized for their ecological roles in attracting pollinators and seed dispersers, anthocyanins are now understood to play a significant role in plant stress resistance and photoprotection [1]. Besides their physiological functions in plants, dietary anthocyanins from fruits and vegetables have attracted attention for their strong antioxidant, anti-inflammatory, and metabolic regulatory properties, with implications for cardiovascular health, diabetes management, and cancer prevention [2,3,4]. However, questions about their bioavailability and optimal intake remain unresolved [5,6].

Anthocyanin biosynthesis is orchestrated through a well-characterized metabolic pathway, culminating in glycosylated anthocyanin derivatives. This pathway is tightly regulated by MYB-bHLH-WD40 (MBW) transcriptional complexes, which modulate gene expression in response to developmental cues and environmental stimuli [7]. Among these stimuli, light serves as a pivotal regulator of anthocyanin accumulation. Plants have evolved intricate signaling networks to perceive fluctuations in light quality, intensity, and duration, involving photoreceptors such as UVR8, cryptochromes, and phytochromes, and downstream transcriptional regulators including HY5, COP1, and MBW complexes [8,9,10].

The E3 ubiquitin ligase COP1 (CONSTITUTIVE PHOTOMORPHOGENIC 1) plays a central role in light/dark signaling by targeting transcription factors for degradation via the 26S proteasome in the dark, thereby repressing anthocyanin biosynthesis [11]. Upon light exposure, COP1 is excluded from the nucleus, relieving repression and enabling transcriptional activation of anthocyanin biosynthetic genes [12]. In the model species *Arabidopsis thaliana*, the bZIP transcription factor HY5 (ELONGATED HYPOCOTYL 5) is a well-established COP1 substrate [13]. The *cop1-4* mutant accumulates elevated anthocyanin levels, and analysis of the *cop1-4 hy5-215* double mutant reveals that anthocyanin biosynthesis can proceed via HY5-independent mechanisms [14], suggesting the existence of additional COP1-regulated components, although indirect effects cannot be excluded.

The central role of HY5 in anthocyanin biosynthesis has been documented [9,15,16,17]. However, emerging evidence points to a broader regulatory landscape in which COP1 modulates multiple transcriptional nodes. This review aims to explore the multifaceted role of COP1 in anthocyanin biosynthesis, highlighting alternative regulatory routes and potential targets. A deeper understanding of these points of control may inform strategies to enhance anthocyanin accumulation in diverse crop species.

## 2. Anthocyanin Benefits for Plants and Human Health

Anthocyanins are water-soluble flavonoid pigments whose coloration and biological activity are governed by the flavylium cation core structure [18]. Among the six most prevalent anthocyanidins (cyanidin, delphinidin, malvidin, peonidin, petunidin, and pelargonidin), cyanidin 3-*O*-glucoside and cyanidin 3-*O*-rutinoside are particularly abundant and widely distributed in nature [19]. These compounds are responsible for the vibrant pink, red, purple, and blue hues of a variety of plant tissues, including leaves, flowers, and fruits, many of which are consumed as part of the human diet [20].

Anthocyanins are bioactive, non-essential dietary components with health-promoting properties that extend beyond their role as natural colorants. Their concentration in plant tissues is modulated by genetic background, environmental conditions, and developmental stage. Rich dietary sources include red and purple berries, grapes, apples, plums, and red cabbage, with berries, particularly blueberries and blackberries, recognized as among the most concentrated sources [21]. To date, over 1000 distinct anthocyanin structures have been identified, underscoring their structural diversity and therapeutic potential [20].

The health benefits associated with anthocyanin consumption are well documented and include enhanced vascular function [22], improved glycemic control and diabetes prevention [23,24], anti-inflammatory activity [25,26], neuroprotection [18], and cancer prevention [2,26]. These effects are primarily attributed to their antioxidant mechanisms, which encompass free radical scavenging, activation of endogenous antioxidant enzymes, and metal ion chelation [27]. The efficacy of these mechanisms is closely linked to anthocyanin concentration and chemical structure.

As evidence for their health-promoting effects continues to grow, anthocyanins are increasingly recognized as valuable dietary components. Their widespread occurrence in fruits and vegetables makes them an accessible and safe strategy for disease prevention and health maintenance.

In addition to their relevance in human nutrition, anthocyanins play critical roles in plant physiology. They are among the most prominent specialized metabolites, contributing to oxidative stress mitigation, reactive oxygen species (ROS) scavenging, UV protection, and enhanced growth under adverse environmental conditions [28,29,30,31]. Their pigmentation also facilitates ecological interactions by attracting pollinators and seed dispersers [32]. Anthocyanins fulfill diverse ecological functions that have contributed to their evolutionary persistence. Their roles in photoprotection and plant-animal interactions underscore their adaptive significance. The dynamic regulation of anthocyanin biosynthesis in response to environmental cues provides plants with a versatile survival strategy [33].

Under abiotic stress, plants frequently upregulate anthocyanin biosynthesis as part of their adaptive response. Elevated anthocyanin levels bolster antioxidant defenses and stabilize cellular structures, improving resistance to drought, salinity, temperature extremes, and heavy metal exposure [1,34]. These compounds have also been suggested to enhance photosynthetic efficiency under high light conditions and mitigate nutrient stress, particularly under phosphorus-deficient environments [35].

Given their dual importance in promoting human health and enhancing plant resilience, elucidating the regulatory networks that control anthocyanin biosynthesis may facilitate the development of anthocyanin-enriched crops through conventional breeding or metabolic engineering.

## 3. The General Anthocyanin Biosynthesis Pathway

**Figure 1:**
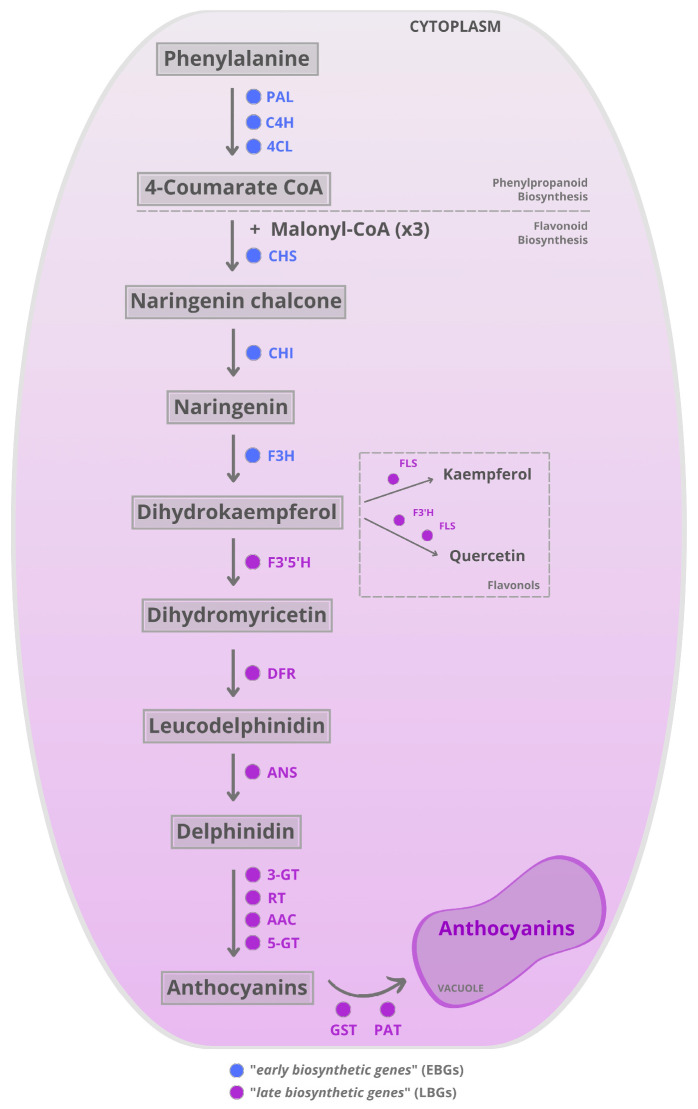
Anthocyanin biosynthesis pathway and vacuolar sequestration involves the coordinated enzymatic steps and transport, including early biosynthetic genes (EBGs, in blue font): phenylalanine ammonia-lyase (PAL), cinnamate 4-hydroxylase (C4H), 4-coumarate:CoA ligase (4CL), chalcone synthase (CHS), chalcone isomerase (CHI), flavanone 3-hydroxylase (F3H), followed by the late biosynthetic genes (LBGs, in magenta font): flavonol synthase (FLS), flavonoid 3′-hydroxylase (F3′H), flavonoid 3′,5′-hydroxylase (F3′5′H), dihydroflavonol 4-reductase (DFR), anthocyanidin synthase (ANS), UDP-glucose:flavonoid 3-*O*-glucosyltransferase (UFGT), anthocyanin 5-*O*-glucosyltransferase (5-GT), glutathione S-transferase (GST), and a putative anthocyanin transporter (PAT).

Anthocyanin biosynthesis is governed by a coordinated network of structural and regulatory genes that respond dynamically to developmental signals and environmental stimuli. The pathway initiates with the deamination of phenylalanine to cinnamic acid, catalyzed by phenylalanine ammonia-lyase (PAL), followed by hydroxylation via cinnamate 4-hydroxylase (C4H) and activation by 4-coumarate:CoA ligase (4CL), yielding 4-coumaroyl-CoA. This intermediate enters the flavonoid biosynthetic route, where chalcone synthase (CHS) condenses it with three malonyl-CoA units to produce naringenin chalcone. Chalcone isomerase (CHI) then facilitates stereospecific cyclization to form naringenin, which is hydroxylated at the C-3 position by flavanone 3-hydroxylase (F3H), generating dihydroflavonols. These steps are catalyzed by enzymes encoded by early biosynthetic genes (EBGs), which comprise genes involved in the general flavonoid pathway (Figure 1) [7]. Subsequent reactions are mediated by late biosynthetic genes (LBGs), which are specifically responsible for anthocyanin formation. Dihydroflavonols undergo further hydroxylation by flavonoid 3′-hydroxylase (F3′H) and flavonoid 3′,5′-hydroxylase (F3′5′H), which determines the hydroxylation pattern of the B-ring and influences anthocyanin coloration. The final steps involve dihydroflavonol 4-reductase (DFR), anthocyanidin synthase (ANS), and UDP-glucose:flavonoid 3-*O*-glucosyltransferase (UFGT), which convert dihydroflavonols into anthocyanidins and subsequently into stable glycosylated anthocyanins [7]. At the dihydroflavonol node, flux can also be diverted toward flavonol formation via flavonol synthase (FLS), competing with anthocyanin production. Additional glycosylation steps (e.g., 5-*O*-glycosyltransferases, 5-GT) further diversify and stabilize anthocyanins. Anthocyanin biosynthesis occurs in the cytosol, and then these pigments are transported to the vacuole for storage. This transport is mediated by glutathione S-transferase (GST) and/or a putative anthocyanin transporter (PAT), where anthocyanins are stored [36] (Figure 1).

Transcriptional regulation of anthocyanin biosynthesis is primarily mediated by the MYB–bHLH–WD40 (MBW) complex, a conserved regulatory module across plant species. This ternary complex comprises R2R3-MYB transcription factors with dual DNA-binding domains conferring gene specificity, basic helix-loop-helix (bHLH) proteins that enhance DNA binding and complex stability, and WD40 repeat proteins that function as scaffolding elements [37,38].

In *Arabidopsis*, key MYB activators include PAP1 (MYB75), PAP2 (MYB90), and MYB113, which upregulate anthocyanin biosynthetic genes. Their bHLH partners, TT8, GL3, and EGL3, possess basic DNA-binding regions and helix-loop-helix domains that mediate protein–protein interactions. The WD40 protein TTG1 stabilizes the complex and facilitates its assembly [39].

Once formed, the MBW complex binds to the promoter regions of anthocyanin-related genes by recognizing specific cis-regulatory motifs, activating transcription of both EBGs and LBGs, with particular emphasis on *DFR*, *ANS*, and *UFGT*. In species such as tomato (*Solanum lycopersicum*), the MBW complex may also regulate additional transcription factors, either positively or negatively, to fine-tune anthocyanin biosynthesis [40]. Protein–protein interactions within the complex are mediated by conserved domains, with bHLH proteins serving as molecular bridges between MYB and WD40 components [38]. The activity and stability of the MBW complex are further modulated by developmental cues and environmental factors, ensuring precise regulation of anthocyanin production

## 4. Anthocyanin Biosynthesis Activation Is Mediated by Light

Light represents one of the most influential environmental cues regulating anthocyanin biosynthesis in plants, acting through a diverse array of specialized photoreceptors that allow plants perceive distinct wavelengths and trigger transcriptional programs to modulate pigment accumulation. In addition to orchestrating these transcriptional responses, plants employ photoreceptors sensitive to UV-B, blue, red, and far-red light, and these light qualities further refine regulatory outcomes by modulating the activity of COP1 and its interactions with specific downstream targets, thereby integrating external light signals into the molecular framework controlling anthocyanin biosynthesis.

UV-B radiation (280–315 nm) is particularly effective in inducing anthocyanin biosynthesis across angiosperms [41,42,43], a process mediated by the UV-B-specific photoreceptor UVR8 (UV RESISTANCE LOCUS 8), which undergoes monomerization upon UV-B exposure and translocates to the nucleus. Once inside the nucleus, photoactivated UVR8 forms a high-affinity complex with COP1 through its VP peptide motif and photosensory core, thereby competitively displacing VP-motif-containing substrates such as HY5 from COP1 [10,44,45]. This displacement stabilizes HY5, leading to enhanced transcription of flavonoid and anthocyanin biosynthesis genes, ultimately integrating UV-B perception with the regulation of pigment production. In peach (*Prunus persica*), both UVA and UVB irradiation stimulate anthocyanin accumulation, with combined treatments producing additive effects [42]. Similarly, purple pepper (*Capsicum* spp.) exhibits robust anthocyanin induction under UV-B exposure [43]. In blueberries (*Vaccinium* spp.), UV-B treatment elicits stage-specific responses, suggesting that distinct regulatory mechanisms operate during fruit development and maturation [41].

Blue light also promotes anthocyanin biosynthesis, primarily through signaling pathways mediated by cryptochromes, which are photoreceptors that respond to blue wavelengths and activate transcriptional regulators of the biosynthetic pathway [10,39]. Upon blue-light exposure, cryptochromes (CRY1/CRY2) become photoactivated and bind the COP1/SPA complex in a light-dependent manner via their C-terminal VP motifs, thereby competing with COP1 substrates [46,47]. In red lettuce (*Lactuca sativa*), blue light significantly enhances anthocyanin content, although UV-B remains the more potent inducer [48]. In the purple tomato (mutant *Anthocyanin fruit*, *Aft*), combined exposure to blue light and UV-B yields synergistic effects, resulting in maximal anthocyanin accumulation [49].

Although less potent than UV and blue light, red light also contributes to anthocyanin biosynthesis through phytochrome-mediated signaling, as phytochromes perceive red and far-red wavelengths and integrate light signals to modulate pigment production [10,48]. Specifically, light-activated phytochromes (phyA/phyB) interact with SPA proteins and promote disruption or inactivation of the COP1/SPA complex, thereby preventing COP1 from degrading downstream transcription factors and permitting stabilization of its substrates [50]. The convergence of signals from multiple photoreceptors allows plants to finely adjust anthocyanin biosynthesis in response to complex light environments [43].

Consequently, anthocyanin biosynthesis is highly dependent on light availability. Under dark or low-light conditions, accumulation is significantly reduced, which negatively affects the commercial quality of horticultural crops. Some species that naturally accumulate anthocyanins when exposed to light develop a completely non-pigmented phenotype when the organ develops in dark conditions [8,9,12,51,52,53,54].

## 5. COP1 Is an E3 Ubiquitin Ligase That Regulates Protein Degradation

**Figure 2:**
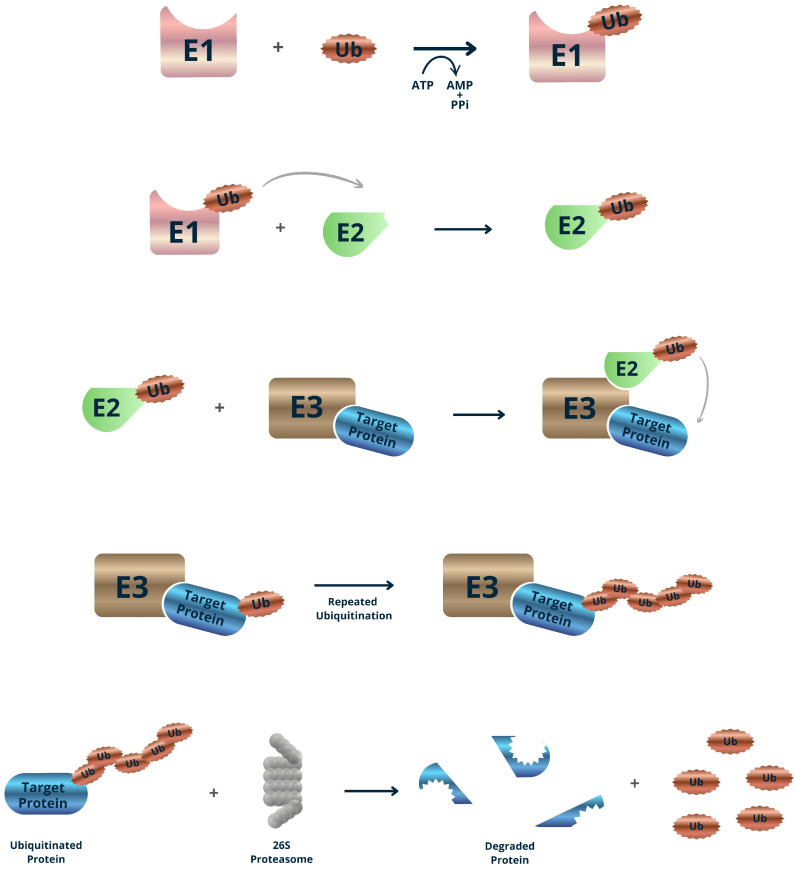
Post-translational regulation mediated by the ubiquitin–proteasome system. Ubiquitination occurs through a three-enzyme cascade that conjugates ubiquitin (Ub) to specific substrate proteins: the E1 ubiquitin-activating enzyme uses ATP to form a high-energy thioester with Ub, Ub is then transferred to an E2 ubiquitin-conjugating enzyme, and an E3 ubiquitin ligase confers substrate specificity by binding both the E2-Ub complex and the target protein to catalyze Ub transfer to a lysine residue on the substrate. COP1 is a RING-type E3 ubiquitin ligase.

Post-translational regulation via the ubiquitin-proteasome system (UPS) is a central mechanism by which plants control protein turnover in response to developmental signals and environmental stress. This system enables selective degradation of proteins, allowing rapid cellular adaptation and reprogramming [11]. The specificity of the UPS is largely determined by E3 ubiquitin ligases, which constitute the most diverse class within the ubiquitination machinery and are responsible for recognizing and targeting substrates for degradation [55]. The UPS operates through a conserved three-step enzymatic cascade involving E1 (ubiquitin-activating enzyme), E2 (ubiquitin-conjugating enzyme), and E3 (ubiquitin ligase). In this process, E1 activates ubiquitin in an ATP-dependent manner and transfers it to E2, forming an E2–ubiquitin conjugate. The E3 ligase then facilitates the transfer of ubiquitin from E2 to specific lysine residues on the target protein, marking it for degradation by the 26S proteasome [56] (Figure 2).

Plant E3 ligases are classified into four major families based on their structural domains and mechanisms of action: RING-type ligases, which contain a RING finger domain that promotes E2–E3 interaction; U-box ligases, which share functional similarities with RING-type ligases but possess distinct structural motifs; SCF complexes (SKP1–Cullin–F-box), which form multi-subunit assemblies for targeted degradation; and HECT-type ligases, which are less common but play essential roles in specific regulatory contexts [57,58]. These ligases are involved in a wide array of physiological processes, including hormone signaling, stress response [11,59], development, light signaling [60], and immune regulation [61,62]. Among them, COP1 is a well-characterized RING-type E3 ligase that acts as a central repressor of light signaling pathways by targeting key transcription factors for ubiquitination and subsequent degradation, thereby playing a pivotal role in modulating photomorphogenic development and anthocyanin biosynthesis. An illustrative example of a multi-subunit RING-type E3 in plants is the COP1/SPA complex, which consists of the RING E3 core COP1 and SUPPRESSOR OF PHYA-105 (SPA) proteins [63,64,65]. SPA proteins function as regulatory cofactors that associate with COP1, thereby enhancing its E3 ligase activity toward specific transcription factor substrates. This interaction provides an additional layer of substrate specificity and facilitates signal integration within light signaling pathways [64,65,66]. In the dark, the COP1/SPA complex mediates the polyubiquitination of selected target proteins, marking them for degradation via the 26S proteasome. Conversely, upon light perception, activated photoreceptors inhibit COP1/SPA activity by disrupting COP1-SPA interaction and/or promoting SPA protein degradation [14,64,67]. This mechanism exemplifies how components of UPS are subject to dynamic regulation in response to environmental cues.

Rather than functioning as a binary dark (nuclear/on) versus light (cytosolic/off) switch, COP1 is now recognized as a highly context-dependent E3 ubiquitin ligase. Its activity is modulated by graded and cell type-specific changes in nucleocytoplasmic partitioning, differential inputs from distinct photoreceptors, and dynamic availability of substrate and cofactor [55,64,65,68].

## 6. COP1 Regulation of Anthocyanin-Related Genes in Different Species

**Figure 3:**
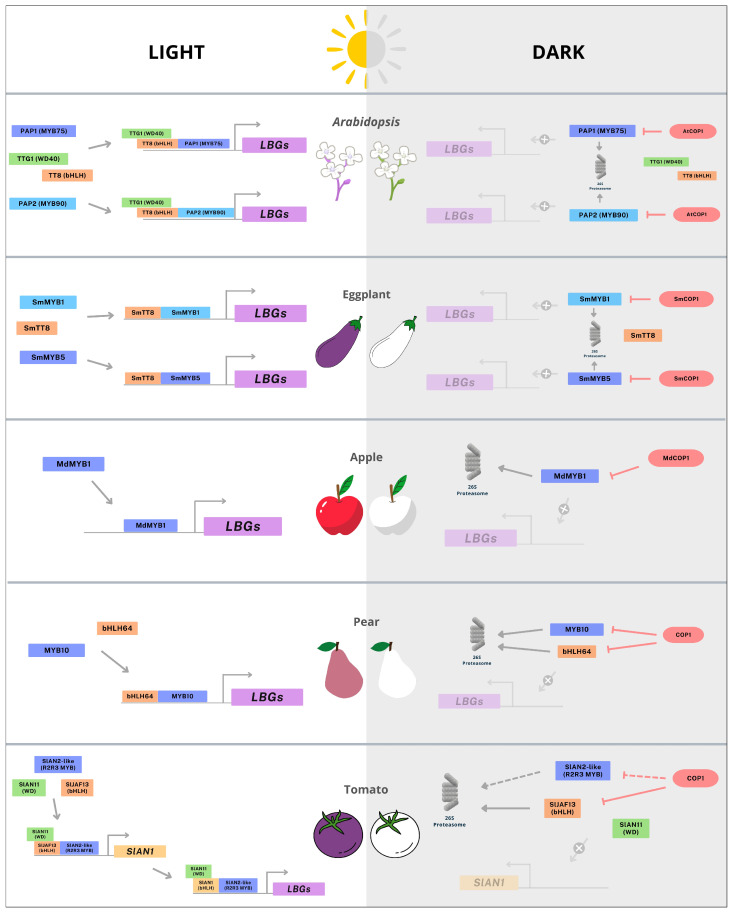
Summary of COP1 regulation via the 26S proteasome, affecting anthocyanin-related genes in *Arabidopsis* [14], eggplant [52,53], apple [12], pear [73,74], and tomato [51,76]. Gray solid arrows indicate activation; red solid lines indicate repression; solid arrows represent experimentally validated mechanisms, whereas dashed arrows and lines indicate possible control mechanisms.

Anthocyanin biosynthesis in plants is tightly regulated by a conserved light-dependent signaling network, in which COP1 functions as a central E3 ubiquitin ligase. Under dark conditions, COP1 targets key transcription factors for ubiquitination and subsequent degradation via the 26S proteasome, thereby repressing pigment accumulation in response to environmental light cues. One of COP1’s primary substrates is HY5, a bZIP transcription factor that integrates light signals and activates anthocyanin-related genes by binding to G-box elements in their promoters [10,41]. Consequently, COP1-mediated HY5 degradation in the dark suppresses anthocyanin biosynthesis [69,70,71]. However, the regulatory role of COP1 extends beyond HY5, as multiple studies have demonstrated its involvement in the degradation of other anthocyanin-related transcription factors, resulting in reduced pigment accumulation [12,14,52,53,72,73,74,75,76] (Figure 3).

The first *Arabidopsis cop1* mutant, identified in the early 1990s, exhibits constitutive photomorphogenic development in the dark, characterized by short hypocotyls, open cotyledons, and light-responsive gene expression in the absence of light [77]. COP1 regulates a broad array of proteins, including HY5, HFR1, CONSTANS, PIFs, DELLA proteins, and notably PAP1 (MYB75) and PAP2 (MYB90), which are key regulators of anthocyanin biosynthesis within the MBW complex [14,78,79,80]. Loss-of-function mutants and RNAi lines targeting *PAP1* exhibit reduced anthocyanin levels, while overexpression of PAP-related MYBs enhances pigment accumulation [81,82]. Importantly, *PAP1* overexpression increases anthocyanin levels only in light-grown seedlings, with no comparable effect in the dark [83]. Despite similar transcript levels, PAP1 and PAP2 proteins accumulate more in light-grown seedlings, indicating post-translational regulation. Co-immunoprecipitation and proteasome inhibition assays confirmed that these MYB proteins interact with COP1 and are degraded in the dark, while light exposure stabilizes them, promoting anthocyanin biosynthesis [14].

A similar mechanism operates in apples (*Malus domestica*), where MdMYB1 is the principal R2R3-MYB transcription factor driving anthocyanin biosynthesis [84,85]. Although *MdMYB1* transcript levels are higher in light-exposed tissues, protein accumulation is suppressed in the dark due to degradation mediated by MdCOP1-1 and MdCOP1-2, homologs of *Arabidopsis COP1*. These homologs physically interact with MdMYB1 and promote its ubiquitination and proteasomal degradation. Functional complementation of the *Arabidopsis cop1-4* mutant with *MdCOP1s* restored photomorphogenic traits, confirming their functional equivalence. Overexpression of *MdCOP1s* in apple fruit tissues reduced pigmentation, while silencing enhanced coloration, without altering *MdMYB1* transcript levels [12], highlighting a conserved post-translational regulatory mechanism.

In eggplant (*Solanum melongena*), anthocyanin biosynthesis is strongly light-dependent, especially in cultivars such as ‘Lanshan Hexian’. Fruits grown in the dark develop a white phenotype lacking anthocyanins, while exposure to sunlight restores pigmentation. SmMYB1 and SmMYB5 are key transcription factors in this pathway and are targeted by SmCOP1 [52,53]. Under light conditions, *SmMYB1* expression is upregulated, whereas *SmCOP1* is more abundant in dark-grown fruit peel. Protein interaction studies confirmed that SmCOP1 promotes SmMYB1 degradation via the 26S proteasome, suppressing anthocyanin biosynthesis [53]. SmMYB5, although light-responsive and independent of SmHY5, also undergoes COP1-mediated degradation in the dark. MG132 treatment confirmed the interaction of SmCOP1 with SmMYB5, reinforcing the role of light in stabilizing anthocyanin-promoting proteins [52].

Further evidence from transgenic eggplant lines with RNAi-mediated silencing of *SmCIP7*, an ortholog of *Arabidopsis COP1-Interacting Protein 7* (*AtCIP7*), revealed reduced anthocyanin pigmentation accompanied by transcriptional reduction of the bHLH transcription factor *SmTT8* [72]. Although SmCIP7 does not directly interact with SmTT8, it may modulate COP1-mediated ubiquitination of transcription factors that regulate SmTT8, suggesting a positive regulatory role in anthocyanin biosynthesis.

In pear (*Pyrus* spp.), COP1 also acts as a negative regulator. In the ‘Red Zaosu’ variety of red pear (*P. pyrifolia*), fruits grown in the dark remain unpigmented, but exposure to blue light induces anthocyanin accumulation. Protein-protein interaction assays demonstrated that PpCOP1 interacts with PpMYB10, and two *COP1-like* genes, *PbCOP1.1* and *PbCOP1.2*, were identified in Chinese pear, with expression inversely correlated with anthocyanin levels [73,75]. Overexpression of these genes suppressed pigmentation, while the bHLH transcription factor PpbHLH64, which interacts with PpMYB10 to activate *PpUFGT* expression, was shown to be degraded in the dark via COP1-mediated proteolysis [74], indicating that bHLH proteins are also COP1 targets.

In tomato, anthocyanin biosynthesis involves a two-step MBW complex formation. SlJAF13, a bHLH transcription factor, forms the first MBW complex with SlAN2-like (MYB114/ANTHOCYNIN FRUIT) and SlAN11, activating *SlAN1* expression. SlAN1 then participates in the second MBW complex to activate structural genes [40]. Although *SlJAF13* is constitutively expressed, its protein levels increase under light and decrease in the dark, suggesting post-translational regulation. SlCOP1 interacts with SlJAF13 and promotes its degradation via the proteasome, as confirmed by MG132 treatment and overexpression assays [76]. *SlCOP1* expression is elevated in non-pigmented fruit tissues and may regulate SlAN2-like protein stability, although further validation is required [51].

Across these five species, *Arabidopsis*, apple, eggplant, pear, and tomato, COP1 consistently functions as a negative regulator of anthocyanin biosynthesis by targeting key transcription factors for degradation. While the core mechanism is conserved, in which light inhibits COP1 activity and stabilizes anthocyanin-promoting proteins, species-specific differences emerge in the diversity of COP1 targets and the complexity of their regulatory networks. *Arabidopsis* COP1 regulates a broad spectrum of transcription factors, whereas apple and pear COP1s primarily target MYB and bHLH proteins. In eggplant, COP1 modulates multiple MYBs and SmCIP7, and in tomato, it targets bHLH components of the MBW complex. These findings underscore the evolutionary conservation of COP1-mediated post-translational control and its pivotal role in light-dependent pigmentation across diverse plant species.

Experimental support for COP1-substrate interactions varies substantially across the targets discussed here. Several relationships are underpinned by strong genetic and in vivo evidence, including phenotypic and molecular analyses of *Arabidopsis cop1* mutants, functional complementation studies in apple, and RNA interference (RNAi) silencing in eggplant. By contrast, other reported interactions are supported mainly by transient expression approaches or in vitro protein-protein assays, such as co-immunoprecipitation in *Arabidopsis*, MG132-based degradation assays in tomato, and protein interaction assays in pear. Collectively, these differences in experimental context and rigor indicate that COP1 targets cannot be regarded as equivalently validated, particularly in crop species where definitive functional evidence remains comparatively limited. Explicitly acknowledging this spectrum of support clarifies which roles of COP1 are well established and highlights key targets that warrant further experimental confirmation.

## 7. Conclusions

Recent advances have significantly deepened our understanding of light-regulated anthocyanin biosynthesis, particularly in relation to transcriptional control. However, post-translational mechanisms remain comparatively underexplored, despite their critical role in fine-tuning pigment accumulation. As highlighted throughout this review, COP1 functions as a conserved negative regulator of anthocyanin biosynthesis under dark conditions across multiple plant species. Importantly, COP1 does not directly target multicomponent transcriptional assemblies such as the MYB-bHLH-WD40 (MBW) complex; instead, its E3 ligase activity is directed toward specific protein substrates. In this context, COP1 promotes the ubiquitination and turnover of individual MYB and bHLH components, thereby indirectly modulating MBW-dependent transcription, as well as other regulators that influence pigment biosynthesis. Although the overall repressive function of COP1 is broadly conserved, both substrate identity and the architecture of COP1-centered regulatory networks vary markedly among species, highlighting the balance between a shared regulatory logic and pronounced species-specific nuances in COP1-mediated control.

This evidence underscores the importance of post-translational regulation as a complementary layer to transcriptional control in the anthocyanin biosynthetic pathway. A comprehensive understanding of anthocyanin regulation requires integration of both transcriptional and post-translational networks, particularly given the dynamic nature of environmental responses and developmental cues. Such integrative knowledge holds substantial promise for crop improvement strategies. By manipulating key regulatory nodes, both at the gene expression and protein stability levels, it may be possible to engineer anthocyanin-enriched cultivars with enhanced stress resistance, improved nutritional profiles, and greater visual appeal. Future research should prioritize the elucidation of these regulatory interactions to enable precise and sustainable modulation of anthocyanin traits in economically important crops.

Notably, the mechanistic evidence summarized here remains taxonomically concentrated in *Arabidopsis* and a limited set of dicot horticultural species. In other plant lineages, including monocot horticultural crops and many woody or medicinal species, COP1 involvement in pigmentation is often inferred from expression patterns or heterologous assays, while direct COP1-dependent post-translational targets in the anthocyanin pathway remain largely untested. Accordingly, a key priority moving forward is to extend comparable, mechanistic validation across a broader phylogenetic spectrum, using standardized in vivo protein stability and ubiquitination assays coupled with targeted genetic perturbation in representative monocots and additional perennials. These efforts will help distinguish conservation of a shared COP1 regulatory logic from lineage-specific rewiring of substrate repertoires and network architecture.

Nevertheless, although evolutionarily conserved COP1 regulatory modules are attractive targets for enhancing anthocyanin accumulation, direct manipulation of COP1 must be approached cautiously. COP1 occupies a central position in light-signaling networks and functions as a master regulator integrating multiple developmental and hormonal pathways. As a result, constitutive suppression of COP1 activity is unlikely to be agronomically neutral and may impose trade-offs in growth, yield, or developmental stability through pleiotropic effects. Accordingly, strategies that rely on spatially or temporally restricted modulation, inducible control, or allele-specific fine-tuning of COP1 activity are more likely to enhance anthocyanin biosynthesis while minimizing unintended consequences. Such interventions will be essential for translating mechanistic insights into practical, field-relevant crop improvement outcomes.

## Data Availability

No new data were created or analyzed in this study.

## Abbreviations

The following abbreviations are used in this manuscript:

4CL	4-coumarate:CoA ligase
AN1	ANTHOCYANIN 1 protein (bHLH transcription factor homologous to TT8)
AN11	ANTHOCYANIN 11 protein (WD40 protein, component of the MBW complex)
AN2	ANTHOCYANIN 2 protein (MYB transcription factor homologous to PAP1/MYB75)
ANS	anthocyanidin synthase
ANS	anthocyanidin synthase (also called LDOX, leucoanthocyanidin dioxygenase)
bHLH	basic helix–loop–helix transcription factor family
C4H	cinnamate 4-hydroxylase
CHI	chalcone isomerase
CHS	chalcone synthase
CIP	COP1-interacting protein (generic term; also used for specific COP1 partners in some studies)
CONSTANS	CONSTANS (CO; photoperiodic flowering regulator, B-box zinc-finger TF)
COP1	CONSTITUTIVE PHOTOMORPHOGENIC 1
DELLA	DELLA proteins (GA signaling repressors; named from conserved DELLA motif)
DFR	dihydroflavonol 4-reductase
E1	ubiquitin-activating enzyme
E2	ubiquitin-conjugating enzyme
E3	ubiquitin ligase
EBGs	early biosynthetic genes
F3′5′H	flavonoid 3′,5′-hydroxylase
F3′H	flavonoid 3′-hydroxylase
F3H	flavanone 3-hydroxylase
GST	glutathione S-transferase (anthocyanin-binding/transport-associated GSTs)
HECT	Homologous to the E6-AP Carboxyl Terminus (E3 ubiquitin ligase domain)
HFR1	LONG HYPOCOTYL IN FAR-RED 1 (bHLH transcription factor)
HY5	ELONGATED HYPOCOTYL 5
JAF13	Jasmonate-associated factor 13 (bHLH transcription factor)
LBGs	late biosynthetic genes
MBW	MYB–bHLH–WD ternary regulatory complex
MG132	carbobenzoxy-Leu-Leu-Leu-al (proteasome inhibitor)
MYB	Myeloblastosis (MYB) DNA-binding transcription factor family
PAL	phenylalanine ammonia-lyase
PAP	PRODUCTION OF ANTHOCYANIN PIGMENT (e.g., PAP1/MYB75; PAP2/MYB90 in *Arabidopsis*)
PAT	putative anthocyanin transporter
PIFs	PHYTOCHROME-INTERACTING FACTORS (bHLH transcription factor family)
RING	Really Interesting New Gene (RING) zinc-finger domain (common E3-ligase type)
RNAi	RNA interference
ROS	reactive oxygen species
SCF	SKP1–Cullin–F-box E3 ubiquitin ligase complex
TT8	TRANSPARENT TESTA 8 (bHLH transcription factor regulating flavonoid/anthocyanin genes)
UFGT	UDP-glucose:flavonoid 3-*O*-glucosyltransferase
UPS	ubiquitin-proteasome system
UVR8	UV RESISTANCE LOCUS 8
WD40	WD40 repeat protein (often a scaffold protein; e.g., TTG1)
